# Patterns of schizophrenia symptoms: hidden structure in the PANSS questionnaire

**DOI:** 10.1038/s41398-018-0294-4

**Published:** 2018-10-30

**Authors:** Jérémy Lefort-Besnard, Gaël Varoquaux, Birgit Derntl, Oliver Gruber, Andre Aleman, Renaud Jardri, Iris Sommer, Bertrand Thirion, Danilo Bzdok

**Affiliations:** 1 Department of Psychiatry, Psychotherapy, and Psychosomatics, RWTH Aachen University, Aachen, Germany; 2 Jülich Aachen Research Alliance (JARA) — Translational Brain Medicine, Aachen, Germany; 3 Parietal Team, INRIA, Gif-sur-Yvette, France; 4 Department of Psychiatry and Psychotherapy, University of Tübingen, Tübingen, Germany; 5 Department of Psychiatry, University of Heidelberg, Heidelberg, Germany; 6 BCN Neuroimaging Center, University Medical Center Groningen, University of Groningen, Groningen, The Netherlands; 7 Univ Lille, CNRS UMR9193, SCALab & CHU Lille, Fontan Hospital, CURE platform, 59000 Lille, France; 8 UMC Utrecht Brain Center Rudolf Magnus, Utrecht, The Netherlands

## Abstract

The clinical presentation of patients with schizophrenia has long been described to be very heterogeneous. Coherent symptom profiles can probably be directly derived from behavioral manifestations quantified in medical questionnaires. The combination of machine learning algorithms and an international multi-site dataset (*n* = 218 patients) identified distinctive patterns underlying schizophrenia from the widespread PANSS questionnaire. Our clustering approach revealed a negative symptom patient group as well as a moderate and a severe group, giving further support for the existence of schizophrenia subtypes. Additionally, emerging regression analyses uncovered the most clinically predictive questionnaire items. Small subsets of PANSS items showed convincing forecasting performance in single patients. These item subsets encompassed the entire symptom spectrum confirming that the different facets of schizophrenia can be shown to enable improved clinical diagnosis and medical action in patients. Finally, we did not find evidence for complicated relationships among the PANSS items in our sample. Our collective results suggest that identifying best treatment for a given individual may be grounded in subtle item combinations that transcend the long-trusted positive, negative, and cognitive categories.

## Introduction

Schizophrenia psychopathology is characterized by variability in several clinical aspects. Three symptom groups are commonly thought to be predominant: positive, negative and cognitive^1,2^.

The investigation of the pathophysiological processes leading to schizophrenia symptoms involves the use of standardized rating scales. Various psychological instruments were proposed to quantitatively describe schizophrenia phenomenology. Such clinical assessment tools include the *Scale for the Assessment of Negative Symptoms*^3^, the *Negative Symptom Assessment*^4^, the *Scale for the Assessment of Positive Symptoms*^5^, the *Schedule for Deficit Syndrome*^6^ and the *Brief Psychiatric Rating Scale*^7^. These questionnaires were mostly developed to assess two major dimensions of the psychopathology: the positive and negative syndromes^3,5,8–10^. However, psychometric standardization has not been attested to most of these assessment scales^11,12^. The same goes for the validity of these clinical assessment tools, including the inter-rater reliability, the assessment that the scale's score is not influenced by confounds of no interest, and the coherence of its construction.

In particular, Kay and colleagues^13^ have developed the *Positive And Negative Syndrome Scale* (PANSS) to increase the measure's replicability and objectivity as well as enable direct comparison between positive, negative and more general symptom facets (i.e., cognitive, mood, motor and thought process abnormalities symptoms). The PANSS consists of 30 items. Each item is rated on a seven-point severity scale. The authors have categorized the symptoms into three dimensions. The first two item dimensions capture the positive and negative syndromes consisting of seven different items each. The 16 other items constitute the third item dimension to grasp the general psychopathology. The ensuing instrument presents specified interview guidelines and assessment criteria enhancing the objectivity and replicability of the symptom descriptions. Taken together, the specified PANSS interview allowed enhancing inter-rater reliability, while the inclusion of a third dimension facilitates comparison to other mental disturbances. These added features may explain why the PANSS is today among the most widely used psychometric tools for the evaluation of schizophrenia symptoms.

Despite its widespread adoption, the structure of the PANSS questionnaire is a topic of ongoing debate. The current version of the PANSS questionnaire comprises three subscales. Yet, using principal component analysis (PCA) approaches, several authors suggested different subscales that regroup covarying questionnaire items may yield a better description of heterogeneous schizophrenia symptoms^14–16^. Such quantitative findings have revealed a complex and often inconsistent picture of how the PANSS questionnaire subscales might describe psychiatric patients. For instance, Daneluzzo and colleagues^15^ advanced a three-subscale subdivision of schizophrenia symptoms, whereas Kay and Sevy^17^ reported a solution with seven subscales. Nevertheless, most studies proposing alternative subdivisions of the PANSS have reported five-subscale solutions^18^. In other words, the collection of previous studies revisiting the PANSS provides convincing evidence for the potential of various alternative conceptualizations of schizophrenia symptom dimensions.

To develop and improve symptom scales, factor-analysis procedures were an important statistical tool^19^. In psychology, such multivariate techniques for identifying sources of variation are often applied in the construction of multi-scale questionnaires to determine many-to-many mapping of which items belong to which degree to which scales. Regarding the study of the PANSS questionnaire, PCA was applied for more than half a century to explore the underlying organization of the PANSS questionnaire. Yet, the strong assumptions underlying PCA (i.e., orthogonality) may for instance preclude identification of other rich candidate descriptions of capturing symptoms constellations of a given patient with schizophrenia. Therefore, we hypothesized that the inconsistencies in the previous questionnaire analyses can be reconciled by expanding the repertoire of previously used statistical tools.

Our comprehensive analytic strategy emphasized prediction performance and thus clinical relevance. We used approaches that concentrate on prediction to find generalizable predictive patterns which could enable improvements of clinical workflows. The present investigations thus extended previous research in three ways: First, we more comprehensively explored the underlying organization of the PANSS questionnaire. Second, we focused on the predictability of questionnaire item at the level of single individuals. Third, we charted the possibility of higher-order relationships among questionnaire items. Combined with benefits of using a large data set, this analysis framework offers a more complete understanding of the underlying form and clinical predictability of the commonly used PANSS questionnaire.

## Methods

### Data resources

**Fig. 1:**
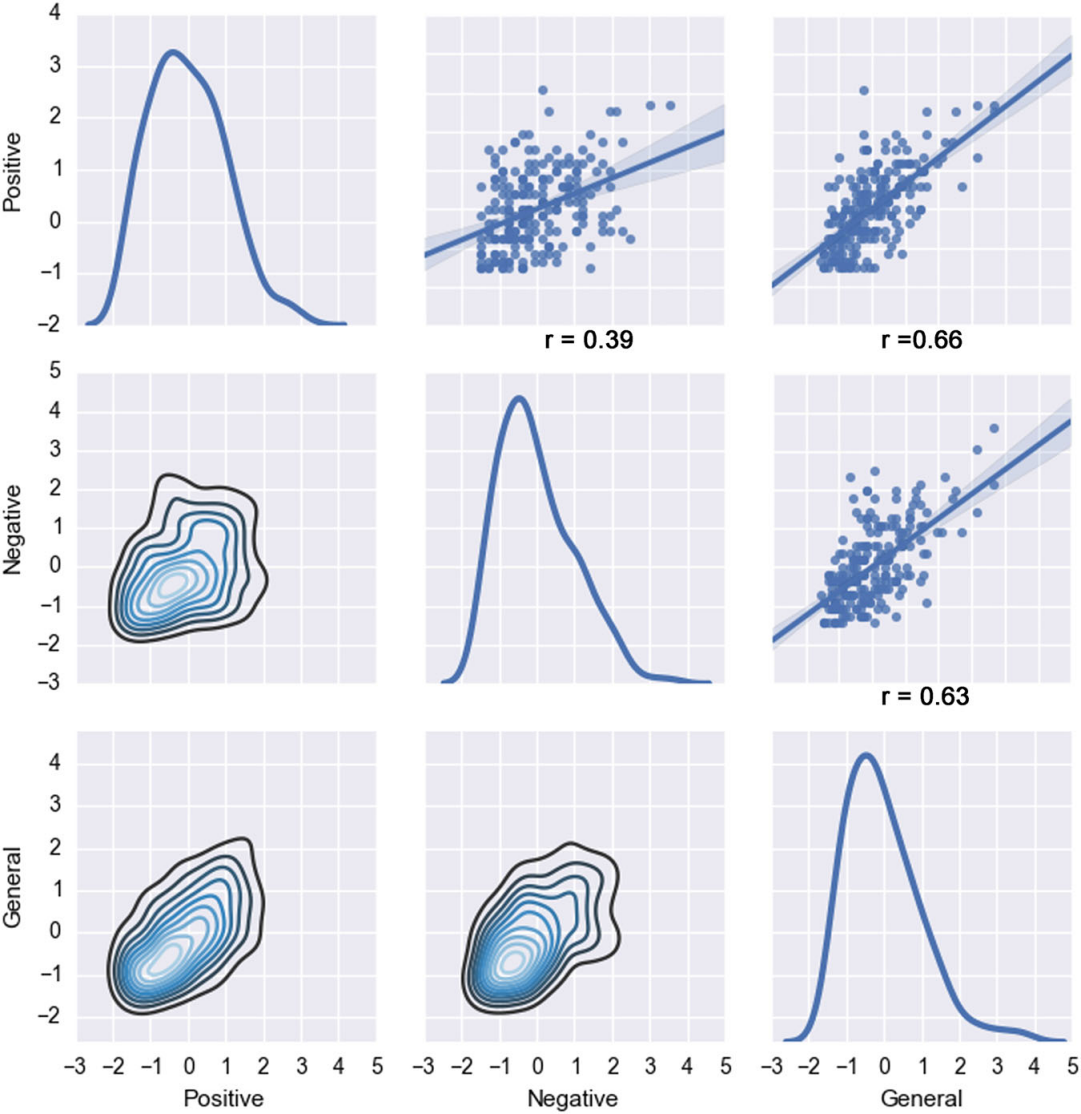
Distribution of questionnaire responses by PANSS categories. In the examined schizophrenia patients, the item scores were summarized by the positive, negative, and general symptom groups structuring the questionnaire. That is, we plotted the standardized items scores mean of each symptom group (positive, negative, and general). Diagonal: the curves represent the individual distribution of the positive, negative and general symptoms items scores. Top-right: the three scatter plots display the linear dependencies between the positive and the negative, the positive and the general, and the general and the negative symptoms items scores and the linear regression of the data sample (with the correlation coefficient *r* noted below). Lower-left: plots the density estimates between each variable. Item responses were *z*-scored to put them on a same par for comparability

We revisited the underlying structure of the PANSS questionnaire based on behavioral data from eight different schizophrenia samples acquired in Europe and the USA (see Supplementary Table 1 for details). The behavioral assessments were collected from a total of 218 patients, including 154 males and 64 female subjects. The distribution of the PANSS questionnaire responses in our sample was homogeneous (Fig. 1).

### Identifying the hidden item stratification: principal component analysis

PCA is the most commonly applied data-analysis method that was previously used to discover hidden factors of variation in the PANSS questionnaire. The majority of previous studies revisiting the PANSS reported five-component solutions. We hence compared the similarities between the five PCA directions extracted from our patient sample and the five latent components found in other psychiatric populations^20–22^.

### Identifying hidden group structure: k-means clustering

We applied a k-means clustering algorithm to automatically partition patient symptom profiles into homogeneous groups. In contrast to PCA, k-means is a method identifying one-to-many mappings^23^: each patient is a member of exactly one group. We used "NbClust"^24^, an established R package that simultaneously applied 30 cluster validity metrics. This approach provided complementary indications of the number of groups most supported by the patient data. Among all indices (using the method “median”) and according to the majority rule, the best number of clusters was 3. That is, the most robust three groups were expressed in the final clustering solution. Therefore, three patient groups of distinct symptom profiles were automatically extracted as it provided a useful fit to our clinical sample.

### Identifying predictive structure: sparse logistic regression

The goal of PCA and k-means was to discover interesting symptoms patterns as measured by PANSS items such as underlying structure and relationship among schizophrenia patients. Complementing these insights in a next step, we applied a modeling technique that emphasizes both prediction performance and automatic identification of the most relevant items.

To achieve this goal, we capitalized on the pattern-learning algorithm *sparse logistic regression*^25^. The sparsity constraint was imposed in form of an *l*_1_ regularization. Such a constraint in the optimization objective automatically detects relevant features “on-the-fly” during model estimation. The *l*_1_ penalty term, calibrated by the hyper-parameter *λ*, is designed to control the parsimony criterion and its shrinkage regularization on the learned model weights. This penalized (negative) log likelihood of the logistic regression objective is given by:

$$- \frac{1}{N}\mathop {\sum }\limits_{i = 1}^N log\left( {1 + e^{ - y_if\left( {x_i;\beta _0,\beta } \right)}} \right) + \lambda \left\| \beta \right\|_1$$

where *x*_*i*_ represents a given patient’s PANSS scores, *y*_*i*_ is his/her schizophrenia severity group defined as the median-split of his/her PANSS total score (0 as mild, 1 as severe), *β*_0_ is the intercept, and *β* is the weight attached to each questionnaire item, the right part of the equation corresponds to the *l*_1_ penalty term controlled by the hyper-parameter *λ*. The item selection behavior depends on the choice of this tuning parameter^26^. The hyper-parameter selection was based on the data in a principled fashion using nested cross-validation. In a common grid of candidate parameter choices, the value of *λ* was varied logarithmically from 3.5 to 1.0 in log-space with 16 steps. The member in the model family that yielded highest prediction accuracy (i.e., generalization performance) for each candidate of *λ* was selected. In other words, the goal here was not to select the best hyperparameter. Rather, we charted a space of candidate *λ* to explicitly investigate the transition from low to high sparsity. In this way, the quantitative investigation detected subsets of items that were most predictive for schizophrenia severity. Finally, we further detailed this analysis with an examination of the learning curve to assess the predictive model performance as a function of increasing sample size.

### Testing for complex relationships among the PANSS items

The k-means method (cf. above) extracted latent structure dormant in the data regardless of symptom severity measures. Sparse logistic regression (cf. above) in turn selected the most predictive variables but this predictive algorithm was not convenient to uncover hidden non-linear relationships between the questionnaire items. We combined exploration of more sophisticated item-item relationships with evaluating prediction performance using non-linear predictive algorithms. In this way, we tested the hypothesis of existing higher-order relationships between the PANSS responses and their usefulness for prediction. We compared the performance of linear models to the performance of models able to exploit non-linear structure in the questionnaires. We complemented this analysis with accuracy–sample-size examination by computing learning curve for each pattern-learning model. Three linear models (ridge regression, logistic regression, and support vector machine) were benchmarked against three models allowing looking for higher-order interactions (k nearest neighbor, random forest and adaptive boosting; see Supplementary Methods for more details). Again, schizophrenia severity was defined as the median-split of the PANSS total score (0 as mild, 1 as severe) representing a categorical summary of the constituent continuous scores.

### Code availability

All analysis scripts of the present study are readily accessible to the reader online (https://github.com/JLefortBesnard/Panss2018). See Supplementary Methods for more details.

## Results

### Factor-structure identified with PCA

In a preparatory analysis, we replicated results from the most often used statistical approach for latent-factor modeling of the PANSS questionnaire administered to schizophrenia patients (SFig. 1). Our findings from the five-component solution were found to be virtually identical to the previously reported findings in other schizophrenia populations^20–22^.

### Properties of patient groups hidden in PANSS questionnaire

Previous research on dimensional many-to-many PCA directions was complemented by assigning each patient to only one dominant constellation of PANSS items in a one-to-many fashion. Each patient was assigned to one and only one k-means cluster. Patients within a cluster were maximally similar, while patients from different clusters are maximally diverging in their symptom constellation.

**Fig. 2:**
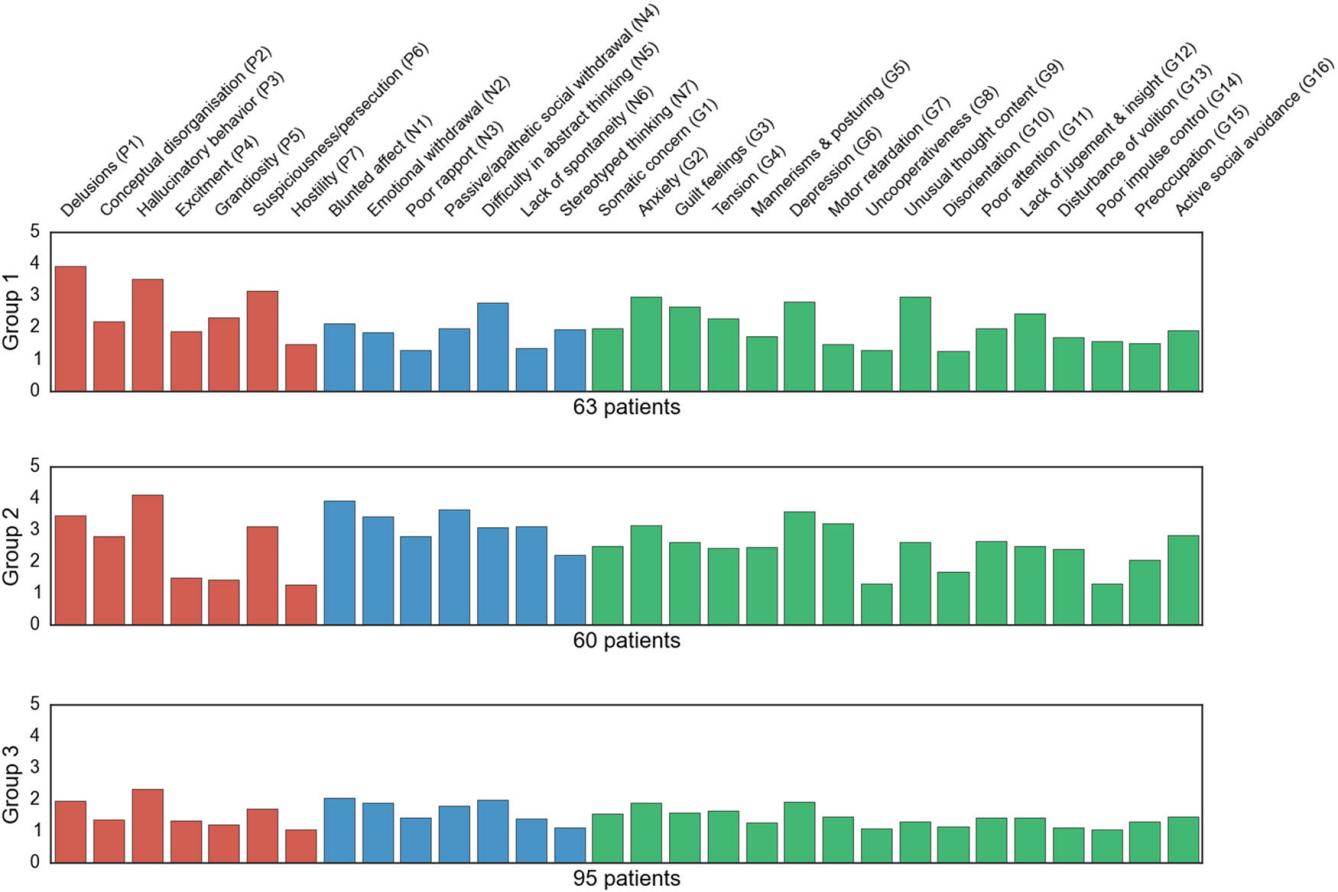
Three patient groups with distinct symptom profiles. Three patient groups were extracted from the data using automatic clustering. Each row represents one data-derived symptom group with a distinct profile of patients. The weights (*x* axis) of each bar (PANSS item on the *y* axis) are automatically determined given the relative importance of the items for a particular group. The red bars are the item scores of the PANSS positive scale in the respective cluster, the *blue* ones are the item scores of the PANSS negative scale and the green bars are the item scores of the PANSS general psychopathology scale. Three different subtypes appeared: a profile including patients with low score for each item (group 3), a profile including patients with very high scores on a number of items related to each type of symptomatology (i.e., negative, positive, and general symptoms, group 1) and a profile including patients scoring very high on items associated with negative symptoms (group 2). In sum, these results suggest a discriminative hidden structure in the PANSS items not only based on a dimensional but also on a categorical aspect of the PANSS

This approach exposed three distinct symptom clusters that grouped the patients: the first group harbored low expression for each questionnaire item, the second group included several quite prominent items scores and the third one displayed a heavy affection on the negative scale (Fig. 2): (i) the first group included patients who scored rather low on most items (maximum 2 points on average). (ii) the second group included patients who scored high (more than 2.5 points on average) on three positive items (delusions, hallucinatory behavior, and suspiciousness/persecution), one negative item (difficulty in abstract thinking) and four general items (anxiety, guilt feelings, depression, and unusual thought content). (iii) The third group included patients scoring high (more than 3 points on average) on three positive items (delusions, hallucinatory behavior, and suspiciousness/persecution), five negative items (blunted affect, emotional withdrawal, passive/apathetic social withdrawal, difficulty in abstract thinking, and lack of spontaneity and flow of conversation), and three general symptom items (anxiety, depression and motor retardation).

Yet, as an exploratory pattern-discovery approach, k-means yield clusters without formal guarantee to offer predictive discriminability between patients that were not part of the present schizophrenia sample^27,28^. A natural next step of the present study therefore consisted in estimating the predictability of schizophrenia severity from PANSS questionnaire items.

### Isolating the most predictive items in the PANSS questionnaire

**Fig. 3:**
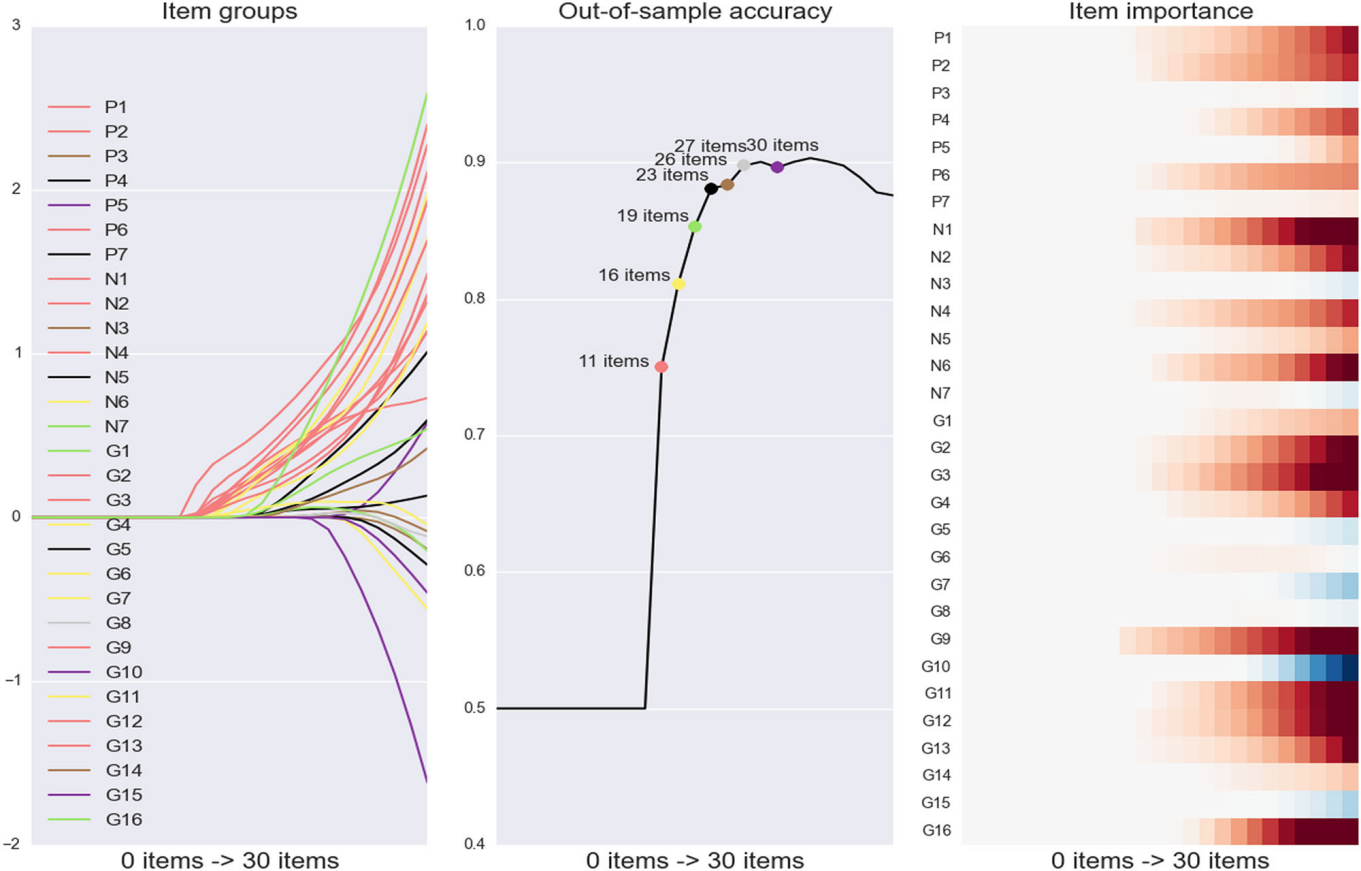
Predictive decomposition of schizophrenia symptom profile. **a** Item groups: a parsimony-inducing learning algorithm was used to search through the array of questionnaire items and extract the most parsimonious subsets of items for predicting schizophrenia severity. Profiles of the classifier coefficients of the PANSS items are plotted on the *y* axis while the decreasing parsimony constraint of this statistical model (here represented as the increasing number of items automatically selected) is plotted on the *x* axis. The departing lines indicate changes in the subset of selected items (i.e., the active set). The color of each line shows the group affiliation of each questionnaire item. **b** Prediction accuracy retraces how prediction performance increases step-by-step as the seven identified item subsets are added to the model. Each colored point represents a predictive model including a specific number of selected items. **c** Relative item importance: item importance in the active coefficients as the parsimony constraint becomes more lenient (left to right). This panel thus represents the relative importance of each item (*y* axis) as more variables are included in the model (*x* axis, from left to right). In sum, the results emphasize that using a model including only eleven PANSS items, schizophrenia severity was predicted with an accuracy only 15% below the accuracy obtained with the model including the 30 PANSS items indicating a very high predictive power for these eleven items

A predictive pattern-learning algorithm (sparse logistic regression) was used to automatically identify item subsets in the PANSS questionnaire that are most informative about telling mild versus severe schizophrenia apart in future patients. The parsimony constraint of this statistical model allowed isolating the most important items to make useful predictions in single psychiatric patients. With systematically varying parsimony constraint, a series of algorithms estimations was carried out to predict schizophrenia severity (defined as the median-split of the PANSS total score) based on the symptom scales (Fig. 3a, c).

Our analysis strategy extracted eleven of the overall 30 items as the most predictive PANSS subset and achieved quite effective prediction of schizophrenia severity (75% accuracy). This essential subset included two items associated with negative symptoms (blunted affect, passive/apathetic social withdrawal), three items associated with positive symptoms (delusions, conceptual disorganization, suspiciousness/persecution), two items associated with emotional symptoms (emotional withdrawal, anxiety), one item associated with social discomfort (guilt feelings), and three items associated with cognitive symptoms (unusual thought content, lack of judgment and insight, and disturbance of volition).

As we relieved the parsimony constraint step-by-step, six other solutions were found that isolated further questionnaire items subsets predictive of schizophrenia severity (Fig. 3b), with 16, 19, 23, 26, 27, and 30 automatically selected items. These subsets reached a prediction accuracy (out-of-sample prediction performance) of 81%, 85%, 88%, 88%, 90% and 90% in new patients, respectively.

As a final step, we analyzed the learning curve to assess the predictive model performance as a function of increasing available sample size (SFig. 2). The performance of the model continuously improved after the training size exceeded 100 patients. This observation suggested that data from more than 100 individuals are beneficial to learn from a powerful predictive model for schizophrenia from behavioral data. The finding also indicated that our multi-site dataset allowed for richer descriptions of the patterns hidden in the PANSS questionnaire.

### Testing for complex relationships among the PANSS items

**Fig. 4:**
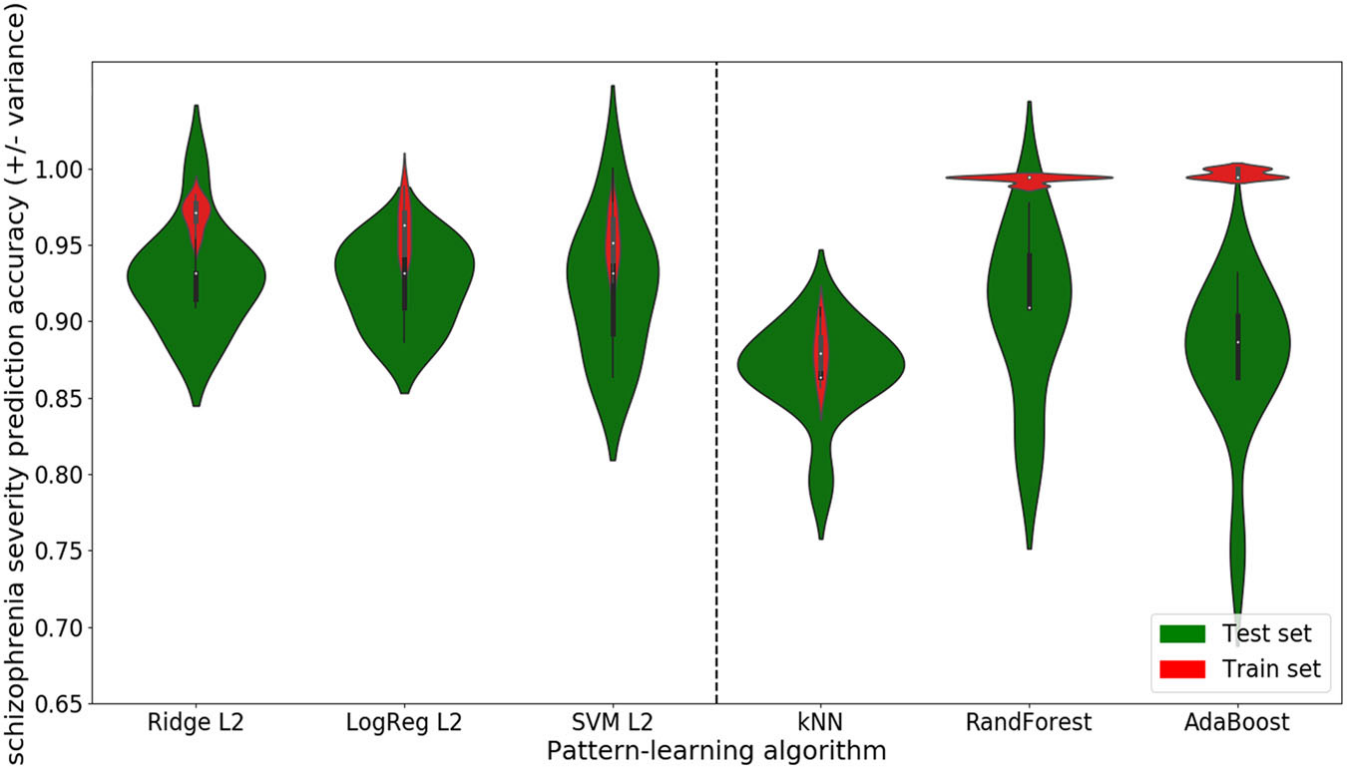
Probing complex relationships among the PANSS items. We explored the hypothesis that more complex patterns may explain relationships between the different PANSS items. We thus compared the predicting performance of models looking for additive effects (left side) to the prediction performance of models looking for higher-order effects (right side). The red violin plots display the in-sample accuracies (train set) while the green plots display the generalization performance (test set). The width of the violins illustrates the density of the obtained performances. For instance, the shape of the first green violin plot on the right side (skinny on each end and wide in the middle) indicates that the obtained accuracies are highly concentrated around the median. The height of the violins indicates the variability (i.e., range of the obtained accuracies). Short violins represent a slight while long violins represent a substantial variability. Linear models including the ridge regression (*Ridge L2*), the logistic regression (*LogReg L2*), and the support vector machine (*SVM L2*) are plotted on the left side of the dashed bar. Non-linear models including the k nearest neighbor (kNN), the random forest (RandForest) and the adaptive boosting (AdaBoost) are plotted on the right side. As a general observation, the green violin plots of the linear models indicate on average a better performance with less variance thus appear to be more adapted to this setting. These results suggest that the PANSS items are perhaps mostly individually predictive as much as this evidence is supported by our multi-site patient dataset

The parsimony-inducing predictive algorithm was a linear model that could only capture how each PANSS item individually contributed to schizophrenia disease, while statistical approaches able to appreciate non-linear structure allow detecting items that together modulate disease severity. To test the hypothesis of existing higher-order interaction between the PANSS items, we compared the prediction performance on schizophrenia severity (defined as the median-split of the PANSS total score) of different linear models (ridge regression, logistic regression, and support vector machine) to the prediction performance of different non-linear ones (k nearest neighbor, random forest, and adaptive boosting) (Fig. 4). Furthermore, we investigated the scaling behavior of each pattern-learning model (SFig. 3).

The three linear models—ridge regression, logistic regression, and support vector machine—obtained on average a better performance with respectively 93%, 92%, and 92% accuracy. Instead, the models looking for higher-order interactions—k nearest neighbor, random forest and adaptive boosting—obtained on average 87%, 91%, and 87%. Furthermore, the variance was higher for the non-linear model performances (average standard deviation: 4.6%) than within the linear model performances (average standard deviation: 3.3%).

Considering the learning curve of the linear models showed that a plateau is reached at 60 patients for two of them (logistic regression and support vector machine) and 130 for the ridge regression giving support to the claim that linear models are proper to extract knowledge in our sample size. On the same line, the k-nearest neighbor training score started to diverge from the test score when given more than 120 patients while the random forest reached a plateau at 130 patients. However, it appears clearly that adaptive boosting keeps on learning and predicting better with more data involved in the fitting.

As a general observation, the linear models predicted more accurately on average with less variance on our patient sample, suggesting that the PANSS items are predominantly predictive for disease severity based on their one-by-one scores. However, this claim might be limited to the size of our sample and increasing the sample size may likely improve the predictive accuracy of non-linear models such as adaptive boosting.

## Discussion

The PANSS questionnaire is pervasively used in psychiatry but has been repeatedly proposed to require revision. We provide a comprehensive characterization for quantifying the different typologies of psychopathology in schizophrenia patients from a multi-site data collection. Furthermore, our study emphasizes the relevance of the questionnaire as behavioral information valuable to effectively predict schizophrenia severity in single individuals. On the one hand, we found that dimensional partitions are candidate fingerprints underlying discrete schizophrenia profiles as it was emphasized in our automatic structure-discovery approaches. On the other hand, an automatic variable-selection algorithm revealed a most important subset of eleven most predictive PANSS items. This quintessential PANSS subset encompassed various parts of the spectrum of schizophrenia symptoms confirming that the different facets of schizophrenia could be shown as useful in robust single-subject predictions for these psychiatric patients. Collectively, our results suggest that some previously inconsistent findings may be reconciled by using an extended repertoire of modern data-analysis tools.

### Extracting predictive subsets of PANSS items

As a primary focus of the present investigation, we automatically identified the most predictive PANSS items of schizophrenia severity. A subselection of the 30 total PANSS items predicted schizophrenia severity with an accuracy of 75% which is only 15% below the accuracy obtained using the full PANSS questionnaire (90%) in our multi-site sample of 218 patients. The eleven items included two items associated with negative symptoms, three items associated with positive symptoms, two items associated with emotional symptoms, one item associated with social discomfort, and three items associated with cognitive symptoms. In sum, this core subset of questionnaire items was highly predictive of schizophrenia severity and presents a quintessential summary of eleven items tapping into parts of the whole spectrum of schizophrenia symptomatology.

Over the past century, the most common practice to understand the risk of developing a mental disorder was to look for the psychopathology's underpinnings by investigating the contribution of each potentially accountable variable to a specific mental disease. However, several advantages arise from predicting behavior (i.e., to accurately forecast behaviors that have not yet been observed) including the ability to test the relevance of existing theories and to discover new mechanisms^29^. Furthermore, a number of successful studies often focused on prediction, rather than placing a premium on scientific discovery. For instance, Koutsouleris and colleagues^30^ used support vector machines to predict the clinical outcomes of individuals in at-risk mental states of psychosis by showing relevance of predictive patterns of whole-brain neuroanatomical abnormalities that could forecast psychosis onset. Recently, Ramsay and colleagues^31^ performed a penalized regression variant and found that global cognition, education, and gender were predictive of improvement on global cognition following a cognitive training in schizophrenia patients, while the explanatory modeling, in the example of Pearson correlation and classical linear-regression-type analyses, did not find any relationship. Therefore, a strategy aiming at prediction appears to be an attractive complementary approach to enable improvements of clinical workflows.

Given the benefit of such analysis framework, we automatically extracted the most predictive items from the PANSS questionnaire. The eleven items highly predictive of schizophrenia severity corroborate results from previous clinical studies. In fact, the five dimensions underlying the psychopathology of schizophrenia often reported in different samples^20–22,32–34^ are comparable with the five symptom domains encompassed by the eleven highly predictive items. This overlap implies that similar underlying determinants of schizophrenia were uncovered by the two complementing approaches (i.e., the explanatory and the predictive strategies). In sum, these extracted symptom constellations, corroborating previous findings, might thus be relevant for further investigations related to disease trajectory.

The PANSS questionnaire is a gold standard for quantifying schizophrenia symptoms but has been repeatedly noted to require further improvement: Indeed, the PANSS questionnaire has been criticized for being lengthy^35^. In fact, 30–40 min are required for the PANSS assessment^13^. Our results show that assessing only a third of this questionnaire may be sufficient for making accurate statements about psychopathological features of schizophrenia patients. This highly predictive subset of PANSS items could help to obtain a fast diagnostic of clinical/psychopathological severity which has several advantages. These benefits include (i) saving clinician time without sacrificing effectiveness, (ii) reducing the time taken for assessing symptoms severity and thereby saving patients time, and (iii) economic advantages such as savings in national health expenditure, increased physician income or reduction of physician work hours.

### Extracting subgroup categories from the PANSS

Using a clustering method, three types of distinct, clinically meaningful symptom categories emerged. A first profile with low expression for each questionnaire item, a second profile with some items scoring really high, and a third profile with a heavy affection on the negative scale. Providing evidence that a major difference between patients is the extent of the negative symptoms, our results also provide support for the possible clinical effectiveness of the subtypes.

Diagnostic manuals such as the DSM and the ICD highlight the focus on ensuring an effective communication of diagnoses between clinicians rather than capturing diagnoses that align well with biological reality. Given that schizophrenia is today widely acknowledged to be a spectrum disorder, modeling schizophrenia intermediate phenotypes (i.e., biological markers) is of great interest. Supplementing discrete disease definition in form of categorical and dimensional additions is an emerging mindset among many clinicians and researchers. Given that clinical subgroups of schizophrenia are often thought as disjoint from each other, we opted for adding “categorical” constraint to the analysis for discovering latent relationships between the PANSS items.

Even though PCA is the most often used statistical framework, a few existing studies also applied a clustering method to extract information from the PANSS. Rolls and colleagues^36^ for instance, also applied a clustering method to a sample of patients diagnosed with schizophrenia. Three types of profile were also identified including a positive and high negative symptoms profile, a positive and intermediate negative symptoms profile and a positive and low negative symptoms profile. Each profile scored high on positive symptoms which was not the case in our study. However, both their results and ours provide evidence that the extent of negative symptoms underlies a major difference between patients. Other authors^37^ also identified three subgroups in another schizophrenia sample identical to the three profiles that became apparent in our sample with in addition a fourth subgroup including patients scoring high only on positive items. Here, unlike our findings, positive symptoms were relevant to distinguish schizophrenia patients. Nonetheless, as in our study, dimensional partitions as well as negative symptoms were found to underlie discrete schizophrenia profiles.

In sum, our results corroborate previous findings suggesting latent structure in the PANSS items mostly based on negative symptom items. Our results have repeatedly emphasized relevance of blunted affect, apathetic social withdrawal, and emotional withdrawal items which were found to be highly predictive of schizophrenia severity in our previous analysis. Moreover, these automatically extracted patient symptom constellations potentially endorse the possibility of existing schizophrenia subtypes.

### Exploring complex patterns in the PANSS

We investigated the idea that more elaborate statistical relationships among questionnaire items may explain the response variability among patients with schizophrenia. We looked for both additive (i.e., linear) and interaction effects. Additive effects imply that the effect produced by two or more symptoms produce a total effect the same as the sum of their individual effects. Interaction effects mean that the combined effect is not additive. In fact, it is widely assumed that higher-order interactions between vulnerabilities triggered by the environment such as growing up in an urbanized area^38^ and vulnerabilities conferred by genes such as NRG1^39^ are important in the etiology of schizophrenia and may result in this major psychiatric disorder^38^. Nonetheless, the very successful genome-wide association studies (GWAS) have been mostly grounded in additive models and thus blind to such interaction effects. In other words, common GWAS applications investigate the separate effect of each individual gene on overall disease vulnerability.

To test the hypothesis of similar interaction effects at the behavioral level as captured by PANSS responses, we compared the prediction accuracy of models looking for additive effects to models able to identify higher-order effects for possible enhanced prediction performance. Furthermore, we detailed this analysis with an examination of each model’s learning curve to assess the predictive model performance as a function of increasing sample size. We found that in our patient sample, PANSS questionnaire items give information about the outcome (i.e., schizophrenia severity) in an additive manner. Indeed, the prediction performance obtained when looking for such additive effects was more consistently higher than when looking for higher-order effects. In sum, the linearity assumption seems to be appropriate given the higher obtained performance as indicated by currently available schizophrenia sample sizes. Nonetheless, our results also suggest that increasing sample size in future studies might be beneficial to extract higher-order effects between items of the PANSS.

Our results emphasizing additive effects between PANSS items, as much as supported by our multi-site patient cohort, have several clinical implications. First, to the best of our knowledge, our study is the first appropriate empirical evidence that validates the strategy of previous research of similar sample size. Indeed, previous PANSS studies have focused on simple effects underlying the questionnaire and our results legitimate this view. Second, our findings support the predictive validity of the extracted subset of highly predictive PANSS items. Finally, these quantitative results suggest that schizophrenia severity is directly proportional to the PANSS questionnaire items. Indeed, such outcomes indicate that simple statistical relationships (e.g., simple correlation) underlie the PANSS items and are sufficient to extract knowledge in such sample size. These simple processes underlying the PANSS evaluation with its relation to schizophrenia severity can be decomposed into parts and reassembled into the same thing easing the interpretation. In sum, emphasizing linear effects underlying the PANSS questionnaire, this exploration endorses our analytic strategy while validating the statistical design of previous PANSS studies.

## Conclusion

Our research exposes a subset of the PANSS items to be highly effective in detecting severe schizophrenia patients. This most predictive fraction of the PANSS items potentially allows for pragmatic, fast and cost-effective early intervention in schizophrenia in a future of precision psychiatry. As another consequence of our findings, identifying the best treatment for a given individual may not be grounded in positive, negative, or cognitive symptoms. Instead, subtle item combinations that transcend these categories may represent a more appropriate focus to better allocate treatment choices to a particular patient.

Schizophrenia, as a highly variable syndrome and major psychiatric disorder, is an important target for personalized medicine. This possible future requires that prevention and treatment strategies should take patient-specific aspects of clinical symptoms into account. Putting a premium on patient group and clinical tool predictability should facilitate procedural streamlining and enhance clinical care and alleviate economic costs. Individualizing treatment can better allocate health-care expenditures for treatments only effective in specific subpopulation of schizophrenia patients. Our results offer new quantitative insights into stratification of schizophrenia populations and might help for the development of improved clinical guidelines and workflows.

## Electronic supplementary material

SUPPORTING INFORMATION APPENDIX
