# Supplementary material for: Patterns of schizophrenia symptoms: hidden structure in the PANSS questionnaire
Source: Transl Psychiatry. 2018 Oct 30;8:237. doi: 10.1038/s41398-018-0294-4 (PMC6207565; doi:10.1038/s41398-018-0294-4)
Supplement: Supplementary file 1 — SUPPORTING INFORMATION APPENDIX [file 41398_2018_294_MOESM1_ESM.docx]

**SUPPORTING INFORMATION APPENDIX**

**"Patterns of Schizophrenia Symptoms:**

**Hidden Structure in the PANSS Questionnaire"**

Jérémy Lefort-Besnard, Gaël Varoquaux, Birgit Derntl, Oliver Gruber, Andre Aleman, Renaud Jardri, Iris Sommer, Bertrand Thirion, Danilo Bzdok

**Contents:**

**1. Supplementary methods (4 pages)**

**2. Supplementary results (1 page)**

**3. Supplementary discussion (1 pages)**

**4. Supplementary figures (3 pages)**

**5. Supplementary table (1 page)**

**6. Bibliography (1 page)**

**1. SUPPLEMENTARY METHODS**

*Data resources*

We revisited the underlying structure of the PANSS questionnaire based on behavioral data from eight different schizophrenia samples acquired in Europe and the USA: Goettingen, Groeningen, Lille, Utrecht, Tuebingen, COBRE, as well as two different samples from Aachen (see Supplementary Table 1 for details). The behavioral assessments were collected from a total of 218 patients, including 154 males and 64 female subjects. Three patients were excluded from the study due to missing items scores in the PANSS questionnaire. The mean age across the eligible participants was 35.3 years (S.D. = 11.42; ranging from 18 to 65), which did not yield a statistically significant difference between males and females (p=0.26). Each subject has been diagnosed by a board-certified psychiatrist in accordance with the clinical criteria of the International Classification of Diseases (ICD-10) or the Diagnostic and Statistical Manual of Mental Disorders (DSM-IV-TR). All patients underwent a 40-45min semi-structured clinical interview with a medical doctor, after which the clinician rated the patient on the 30 PANSS items. The distribution of the PANSS questionnaire responses in our sample was homogeneous (Fig. 1). Ethics approvals were obtained from the ethics committee of each site's university.

*Identifying the hidden item stratification: Principal component analysis*

PCA is the most commonly applied data-analysis method that was previously used to discover hidden factors of variation in the PANSS questionnaire. We therefore applied the same statistical approach to our sample of schizophrenia patients as a point of comparison to previous research.

PCA summarizes the PANSS items scores into a smaller number of representative variables that collectively explain most of the variability across the set of original questionnaire items. The idea is that not all of the 30 PANSS items are equally effective in describing schizophrenia symptoms. PCA seeks weighted combinations of the items that are as interesting as possible, where the concept of *interesting* is measured by the amount that patient scores vary along each emerging dimension. More concretely, PCA explored the relationships between the 30 questionnaire items and exhibits them as a linear combination of uncorrelated variables called *principal components*. Each such component of symptom variation is represented as a set of specific weights for the questionnaire items, one item weight for each hidden variability component. All weights of a given component are typically non-zero in contrast to the sparse modeling approach below. The weights of the first principal component define the most important source of variation that is as close as possible to the patient symptom scores as its direction is indicative of how response profiles vary the most. In other words, the first principal component i) captures a maximum of the information contained in the set of PANSS items, ii) identifies a direction along which the patient scores vary the most, and iii) provides the linear fit that is closest to the patients' clinical profile. The second principal component, in turn, is a linear combination of the PANSS items that is uncorrelated with the first principal component and extracts the largest direction of variance under this constraint. The strategy is analogous for the third and all subsequent extracted components. Each additional principal component must ensure uncorrelatedness to the previous ones while explaining the largest portion of unexplained variance.

The majority of previous studies revisiting the PANSS reported five-component solutions. We hence compared the similarities between the five PCA directions extracted from our patient sample and the five latent components found in other psychiatric populations (1-3).

*Identifying hidden group structure: k-means clustering*

We applied a k-means clustering algorithm to automatically partition patient symptom profiles into homogeneous groups. PCA and k-mean pursued complementary statistical goals in our study, although both these structure-discovery methods simplify the response items into a smaller number of summary scores. While PCA extracts representations of the data explaining most of the variance, k-means finds homogeneous groups among the observed symptom profiles from the patients. Here, the goal was to partition patients into discrete groups so that patients within each group are quite similar to each other in terms of their salient symptoms, while patients in different groups are maximally different from each other. In other words, this common clustering technique automatically establishes k groups that are internally coherent in their presented symptoms but as different as possible from each other. In contrast to PCA, k-means is a method identifying one-to-many mappings (4): each patient is a member of exactly one group. k-means seeks to partition patient symptom profiles into a number of non-overlapping patient groups. k-means requires to prespecify the desired number of groups k. We thus used "NbClust" (5), an established R package that simultaneously applied 30 cluster validity metrics. This approach provided complementary indications of the number of groups most supported by the patient data. Among all indices (using the method "median") and according to the majority rule, the best number of clusters was 3. That is, the most robust three groups were expressed in the final clustering solution. Therefore, three patient groups of distinct symptom profiles were automatically extracted as it provided a useful fit to our clinical sample.

*Identifying predictive structure: Sparse logistic regression*

The goal of PCA and k-means was to discover interesting symptoms patterns as measured by PANSS items such as underlying structure and relationship among schizophrenia patients. Complementing these insights in a next step, we applied a modeling technique that emphasizes both prediction performance and automatic identification of the most relevant items. That is, we wanted to fit a model that i) relates the item scores to the severity of schizophrenia, with the aim of accurately predicting the response for future patient (i.e., out-of-sample prediction) and ii) finds the most parsimonious subsets of predictive PANSS items.

To achieve this goal, we capitalized on the pattern-learning algorithm *sparse logistic regression* (6). On the one hand, this predictive learning algorithm estimates the separating hyperplane (i.e., a linear function) that distinguishes patients with more severe versus more mild schizophrenia symptoms. The outcome y is defined by the severity of schizophrenia. The severity is characterized by the median-split of the PANSS total score (0 as mild, 1 as severe) as it was the categorical summary of the constituent continuous scores. On the other hand, the imposed sparsity constraint identifies a minimal subset of features (i.e., items of the PANSS questionnaire) that is most informative about the differences between patients. While computing a vector of weights associated with the items, similar to linear regression, this approach aims to reduce the weights of items that have little discriminatory value to exactly zero (a feature's weight equal to zero has no effect on the prediction outcome). This procedure results in a subselection of items which have high joint discriminatory power to separate patients with severe versus mild symptoms. In this way, sparse logistic regression extends previous PANSS investigations by automatic *variable selection* (7). Its benefit is rooted in its clear algorithmic definition leading to formal and rigorous choices in automatic variable selection. This additional constraint enforcing parsimony of response items is particularly useful in our study, as it helps pointing to subset of items most predictive of schizophrenia severity. As a result, our modeling findings obtained from the sparse logistic regression are much easier to interpret.

The sparsity constraint was imposed in form of an $\mathcal{l}_{\text{1}}$ regularization. Such a constraint in the optimization objective automatically detects relevant features “on-the-fly” during model estimation. The$\mathcal{l}_{\text{1}}$ penalty term, calibrated by the hyper-parameter λ, is designed to control the parsimony criterion and its shrinkage regularization on the learned model weights. This penalized (negative) log likelihood of the logistic regression objective is given by:

$$-\frac{1}{N}\sum_{i=1}^{N} log\left( 1+e^{-y_{i}f\left( x_{i} ;\beta_{0},\beta\right)} \right)+ \lambda\left\| \beta\right\|_{1}$$

where $x_{i}$ represents a given patient’s PANSS scores, $y_{i}$ is his/her schizophrenia severity, $\beta_{0}$ is the intercept, and $\beta$ is the weight attached to each questionnaire item, the right part of the equation corresponds to the $\mathcal{l}_{1}$ penalty term controlled by the hyper-parameter λ. The item selection behavior depends on the choice of this tuning parameter. Indeed, the sparse logistic regression shrinks the coefficient estimates more toward zero and performs always more aggressive variable selection with increasing λ. The hyper-parameter selection was based on the data in a principled fashion using nested cross-validation. In a common grid of candidate parameter choices, the value of λ was varied logarithmically from 3.5 to 1.0 in log-space with 16 steps. The member in the model family that yielded highest prediction accuracy (i.e., generalization performance) for each candidate of λ was selected. In this way, the quantitative investigation detected subsets of items that were most predictive for schizophrenia severity. In other words, the goal here was not to select the best hyperparameter. Rather, we charted a space of candidate λ to explicitly investigate the transition from low to high sparsity.

Finally, we further detailed this analysis with an examination of the learning curve to assess the predictive model performance as a function of increasing sample size. To this end, we computed the prediction accuracy for the sparse logistic regression for predicting schizophrenia severity in a series of increasing patient subsets for model training.

*Testing for complex relationships among the PANSS items*

The k-means method (cf. above) extracted latent structure dormant in the data regardless of symptom severity measures. Sparse logistic regression (cf. above) in turn selected the most predictive variables but this predictive algorithm was not convenient to uncover hidden non-linear relationships between the questionnaire items. We combined exploration of more sophisticated item-item relationships with evaluating prediction performance using non-linear predictive algorithms. In this way, we tested the hypothesis of existing higher-order relationships between the PANSS responses and their usefulness for prediction. We compared the performance of linear models to the performance of models able to exploit non-linear structure in the questionnaires. We complemented this analysis with accuracy-sample-size examination by computing learning curve for each pattern-learning model. Three linear models (ridge regression, logistic regression, and support vector machine) were benchmarked against three models allowing looking for higher-order interactions (k nearest neighbor, random forest and adaptive boosting). Again, schizophrenia severity was defined as the median-split of the PANSS total score (0 as mild, 1 as severe) representing a categorical summary of the constituent continuous scores.

Among the linear predictive pattern-learning algorithms, the ridge regression is commonly used as a shrinkage method. This model encourages small absolute weights on each item which emphasized the most predictive PANSS items, while linear support vector machines mapped the items as points in space so that items of separate categories (predictive of a mild or a severe schizophrenia) were divided by a maximal gap between the patients.

Regarding the non-linear models, the k nearest neighbor estimator uses the k closest training examples (in our case, the closest patient symptom profiles) in the feature space, while the output is determined by a majority vote across these most similar training examples. In other words, the guessed severity for a given new schizophrenia patient can thus be derived from the schizophrenia severity of the k closest patients in the training set. Further, the random forest algorithm is an ensemble learning method that operates by constructing a multitude of decision trees and outputs its prediction estimate that is the committee decision across all trees. The severity of schizophrenia symptoms in a given patient was thus derived based on the most consistently predicted outcome of the built decision trees. As the last non-linear prediction algorithm, the adaptive boosting algorithm starts by fitting a model on the dataset and then fits additional copies of that model on the same dataset but where the weights of incorrectly judged instances are adjusted such that subsequent fine-tuning of models focusses more on difficult cases.

*Code availability*

Python was selected as scientific computing engine. Capitalizing on its open-source ecosystem helps enhance replicability, reusability, and provenance tracking. Scikit-learn (8) provided efficient, unit-tested implementations of state-of-the-art statistical learning algorithms ([http://scikit-learn.org](http://scikit-learn.org/)). All analysis scripts of the present study are readily accessible to the reader online (https://github.com/JLefortBesnard/Panss2018).

**2. SUPPLEMENTARY RESULTS**

*Comparison to previous hidden-factor investigations*

In a preparatory analysis, we replicated the most often used statistical approach for latent-factor modeling of the PANSS questionnaire administered to schizophrenia patients (SFig. 1). We computed the five dominant components of variation in our multi-site sample using PCA analogous to previous studies (1-3). Our findings from the five-component solution were found to be virtually identical to the previously reported findings in other schizophrenia populations (1-3).

The present PCA decomposition of PANSS items explained 52% of the variance across symptom scales. This result in our sample is similar to what was found in previous studies: 52% (2), 51% (3), up to 57% of variance explained (1). The component most associated with negative symptom items captured the largest amount of the total variance (26%). Several PANSS items were found to be important in both the first component extracted from our dataset and in the first component derived from other patient samples (1-3). This first component was mostly associated with negative symptom items, including disturbance of volition, passive/apathetic social withdrawal, active social avoidance, motor retardation, and emotional withdrawal from the highest to the lowest absolute association. Our second component exhibited most associations with various positive symptom items (10% explained variance). Three items with relevant weights were found both in our component as well as in the component associated with positive symptom items discovered in previous studies (1-3). These items included grandiosity, unusual thought content, and delusions (from highest to lowest weight relevance). The three remaining components explained approximately 5% of the variance each. A component associated with excitement symptoms had two highly weighted items in common with the excitement-related component of previous studies including from the highest to the lowest absolute association poor impulse control and uncooperativeness (1-3). The five items preoccupation, mannerisms and posturing, stereotyped thinking, conceptual disorganization, lack of judgement and insight (ordered from the highest to the lowest absolute weight) were found relevant in our fourth component as well as in previously reported components mostly associated with cognitive symptom (1-3). Finally, the fifth component exposed three relevant items that were also highlighted in the component related to emotional discomfort symptom in previous studies (1-3), including tension, somatic concern, and preoccupation (from the highest to the lowest absolute weight).

In sum, carrying out PCA on PANSS questionnaires from a large schizophrenia sample led to virtually identical directions of variation patterns underlying symptom combinations as in previous factor modeling investigations. Furthermore, the discovered variability components that explained most of the variance prominently included PANSS items associated with negative symptom. Instead, PANSS items from the positive and general scales were more inconsistently emphasized across the components of variation, which is in line with previous findings (1-3).

**3. SUPPLEMENTARY DISCUSSION**

*Extracting continuous axes in the PANSS*

Our investigation set out by replicating the often reported latent-factor results obtained from the principal components of the PANSS questionnaire administered to schizophrenia patients. To evaluate correspondence with a stream of previous PANSS studies, we extracted five most dominant components in our sample. In agreement with previous research, these five directions of variation indicated that symptomatology varies along negative, positive, excitement, cognitive and emotional discomfort symptoms items from the highest to the lowest influence.

Over more than 30 years, insightful information about schizophrenia psychopathology was obtained through the extraction of hidden dimensions in the PANSS questionnaire using an essentially identical statistical tool: principal component analysis. Most studies of clinical behavior have probed item patterns of five overarching components underlying the PANSS questionnaire. An overwhelming majority of previous studies reported a component with relevant loadings on negative symptom items as explaining most of the variance (1-3). Here, we replicated this recurring finding across over a dozen of previous PANSS questionnaire investigations. Indeed, most of the response variability was explained by the component with relevant loadings on negative symptoms items. This first component can be viewed as the direction along which the patient scores are highly variable. Another recurring finding includes a component with relevant loadings on positive symptom items which was also replicated in our sample (1-3). The three other components were also similar to the previous findings as their loadings emphasized variation over an excitement, cognitive, and emotional discomfort syndrome. In this set of preparatory analyses, our results confirmed virtually identical symptom gradients underlying PANSS questionnaire scores suggesting similarities in the underlying properties present in our multi-site dataset and those present in samples from other PANSS investigations.

Analogous to previous studies (1-3), our sample analysis revealed no item associated with positive symptoms that has high loadings in the first component associated with negative symptoms. This observation corroborates previous findings supporting the notion that the negative symptoms represent a source of variation separate from other symptom aspects in schizophrenia. Indeed, across studies, negative symptoms consistently emerged as a separate factor (9). Instead, across previous PCA analyses, PANSS items from the positive and general scales were more inconsistently associated with components of variation (1-3). Explanations for the observed divergences may include that different subgroups of schizophrenia patients differ less systematically in their symptom patterns that are different from the negative symptoms.

In sum, our results are in line with the pentagonal model of representation of PANSS, which defines five hidden factors summarizing schizophrenia patients PANSS scores (3). The pentagonal model includes variation over negative, positive, activation, dysphoric mood and autistic preoccupation symptoms items which mostly corroborate other findings (1, 2) including ours. We indeed obtained five virtually identical directions of variation patterns underlying PANSS questionnaire scores. In other words, we have shown that our sample harbors very similar emerging properties than most of the previously explored schizophrenia patients samples.

**4. SUPPLEMENTARY FIGURES**

**Supplementary figure 1**

**
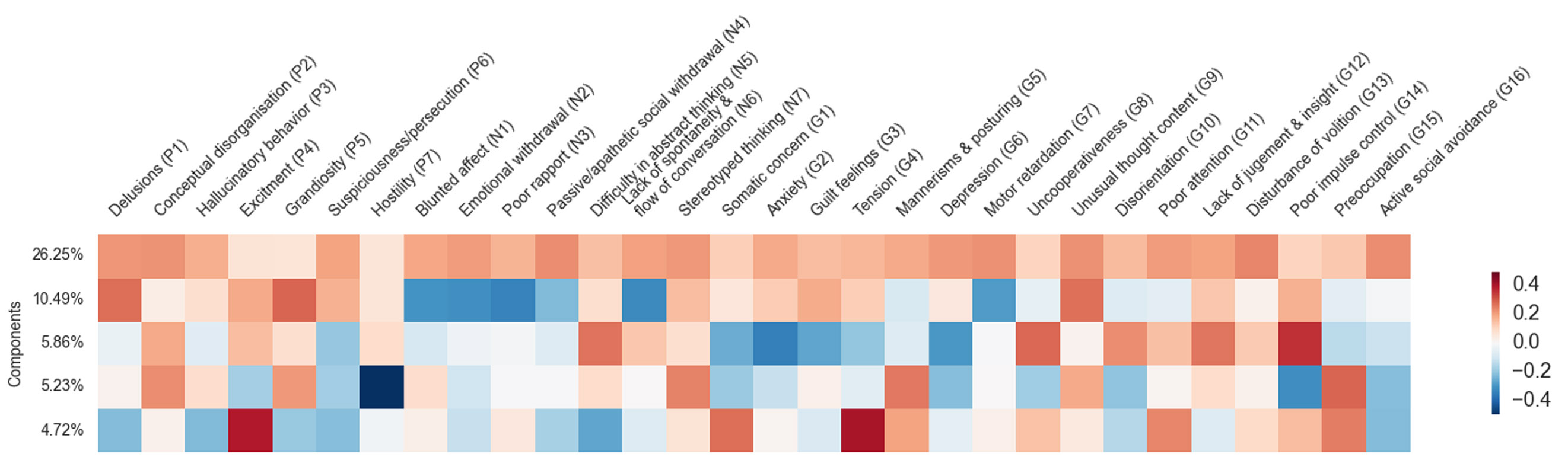
**

**Replication of the previous PCA approaches to decomposing PANSS questionnaire.**

We carry out commonly used principal component analysis to derive five components of variation in the PANSS questionnaire data in our patient sample. Each component and its ratio of explained variance are shown. Each row shows the item relevance (weights) for each component. The findings confirmed earlier descriptions of five components, each closely and specifically associated with a particular schizophrenia symptom including negative, positive, excitement, cognitive and emotional discomfort symptoms. Among these, we obtained one component roughly corresponding to each group of symptoms. Our five-component solution was virtually identical to the components found in a series of previous hidden factor decompositions in schizophrenic patient samples (1-3).

**Supplementary figure 2**

**
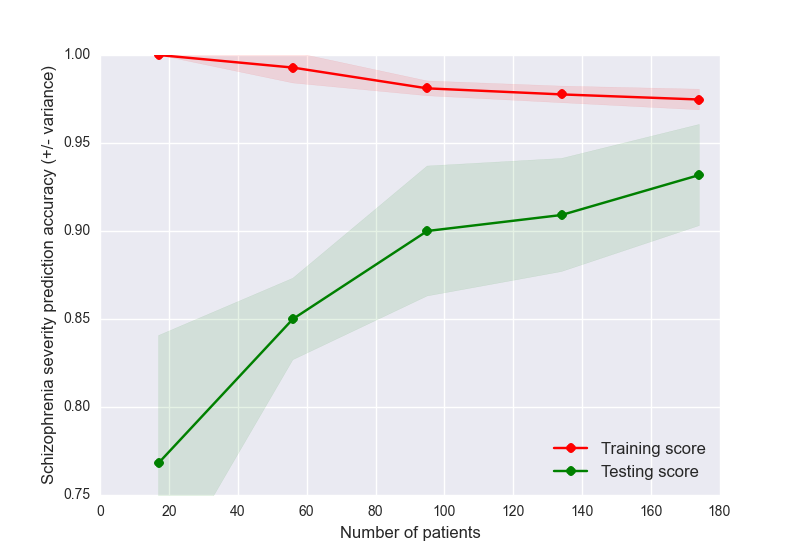
**

**Scaling of schizophrenia prediction accuracy with increasing patients.**

A learning curve is a representation of performance of the learning algorithm with always bigger subsets of the data at hand. The number of patients included is plotted on the x axis while the y axis displays the obtained accuracy for each specific setting (5 splits of the total amount of available data). The *red line* represents the average in-sample (i.e., *training score)* accuracy, while the green line represents the average generalization performance (i.e., *testing score)*. The green and red shadows represent the accuracy standard deviations. Performance in the training set higher than performance in the testing set is to be expected since the training score can dramatically overestimate the test score. Indeed, the learning algorithm learns from the training data and therefore is optimistically biased while the test score represents the efficiency of the model applied to unseen data. While recruitment of more patients would likely further improve the performance of the learning algorithms, we can observe that gathering 90 subjects is a good tradeoff between the needed amount of data and achieving a fair classifier performance.

**Supplementary figure 3**

**
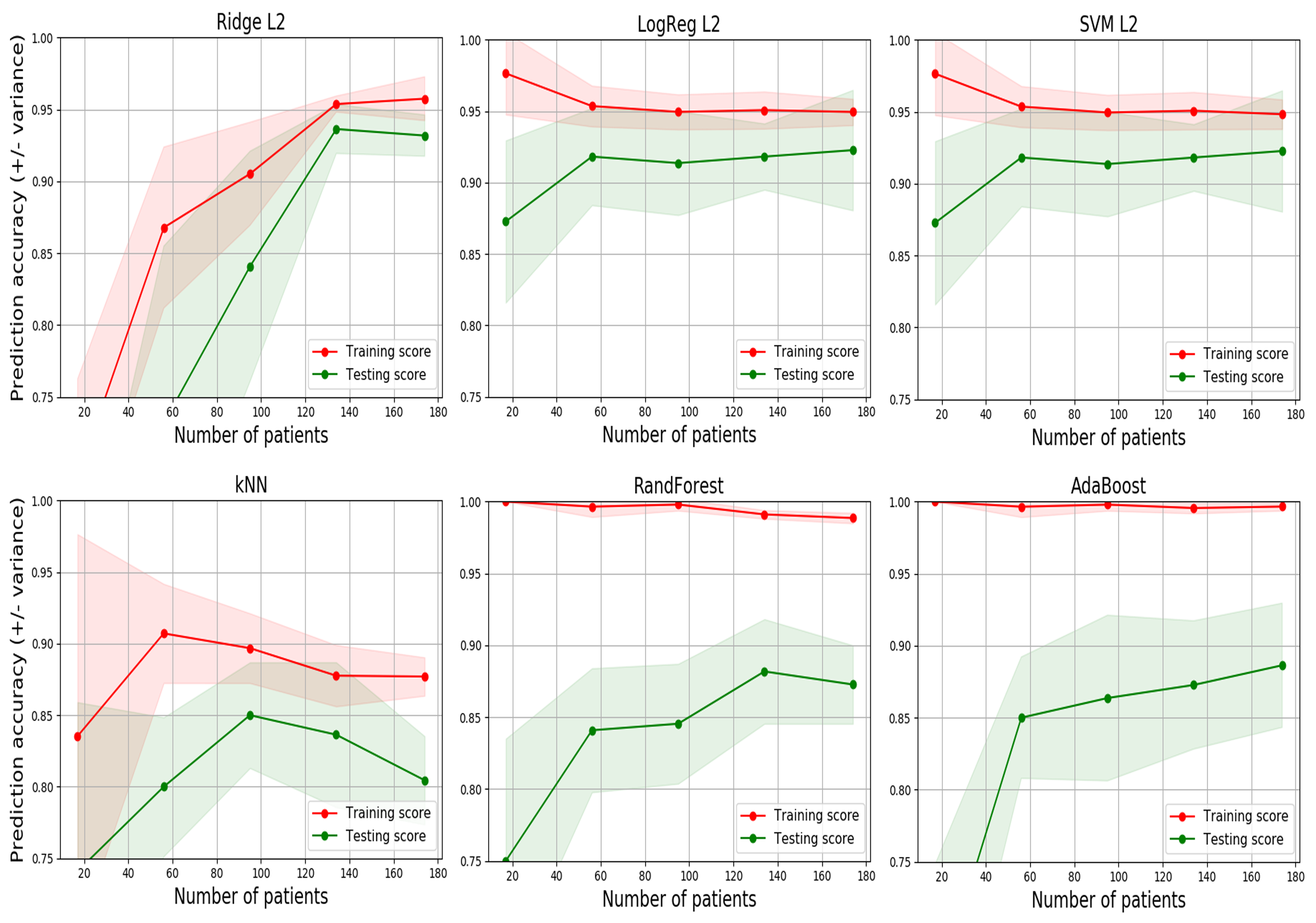
**

**Scaling of linear and non-linear prediction accuracy with increasing patients.**

We explored the learning behavior of each model with always bigger subsets of the data at hand, in the same fashion as the analysis in supplementary figure 2. The learning curve of each linear model (Ridge Regression, Logistic Regression and SVM) is plotted on the first row while the learning curve of each non-linear model (kNN, Random Forest, and Adaptive Boosting) is ploted on the second row. The number of patients whose data is considered is given on the x axis, while the y axis displays the obtained accuracy for each specific setting (5 splits of the total amount of available data). The *red line* represents the average in-sample (i.e., *training score)* accuracy, while the green line represents the average expected generalization performance (i.e., *testing score)*. The green and red shadows represent the accuracy standard deviation. We can observe that gathering around 130 subjects is a good tradeoff between the needed amount of data and achieving a fair classifier performance for both the linear and non-linear models. On the other hand, recruitment of more patients may likely further improve the performance in more data-hungry non-linear models, such as the AdaBoost.

**5. SUPPLEMENTARY TABLE**

**Supplementary table 1:**

| **Sites** | **# patients** | **# male** | **# female** | **Age (M)** | **Age (Std)** | **Age onset (M)** | **Age onset (Std)** |
| --- | --- | --- | --- | --- | --- | --- | --- |
| Aachen | 28 | 20 | 8 | 36,57 | 9,79 | 27,68 | 8,61 |
| Groningen | 32 | 19 | 13 | 32,97 | 11,19 | 25,78 | 9,27 |
| Utrecht | 23 | 13 | 10 | 35,46 | 9,78 | 25,28 | 6,08 |
| Goetingen | 36 | 29 | 7 | 32,06 | 9,81 | 25,61 | 8,16 |
| Tuebingen | 9 | 4 | 5 | 32,11 | 9,37 | 23 | 0 |
| Lille | 18 | 11 | 7 | 33,89 | 7,65 | 22,89 | 2,59 |
| Cobre | 72 | 58 | 14 | 38,17 | 13,89 | 23,61 | 5,95 |
| **TOTAL** | 218 | 154 | 64 | 35,3 | 11,42 | 24,83 | 5,06 |

**6. SUPPLEMENTARY BIBLIOGRAPHY**

1. Bell MD, Lysaker PH, Beam-Goulet JL, Milstein RM, Lindenmayer J-P. Five-component model of schizophrenia: assessing the factorial invariance of the positive and negative syndrome scale. Psychiatry research. 1994;52(3):295-303.

2. Lindenmayer J-P, Grochowski S, Hyman RB. Five factor model of schizophrenia: replication across samples. Schizophrenia Research. 1995;14(3):229-34.

3. White L, Harvey PD, Opler L, Lindenmayer J. Empirical assessment of the factorial structure of clinical symptoms in schizophrenia. Psychopathology. 1997;30(5):263-74.

4. Bzdok D, Yeo BT. Inference in the age of big data: Future perspectives on neuroscience. NeuroImage. 2017;155:549-64.

5. Charrad M, Ghazzali N, Boiteau V, Niknafs A, Charrad MM. Package ‘NbClust’. Journal of Statistical Software. 2014;61:1-36.

6. Hastie T, Tibshirani R, Friedman J. The elements of statistical learning. 2001. 2001.

7. Tibshirani R, Wainwright M, Hastie T. Statistical learning with sparsity: the lasso and generalizations: Chapman and Hall/CRC; 2015.

8. Pedregosa F, Varoquaux G, Gramfort A, Michel V, Thirion B, Grisel O, et al. Scikit-learn: Machine learning in Python. Journal of Machine Learning Research. 2011;12(Oct):2825-30.

9. Blanchard JJ, Cohen AS. The structure of negative symptoms within schizophrenia: implications for assessment. Schizophrenia Bulletin. 2006;32(2):238-45.
